# MRI Texture Analysis in Multiple Sclerosis

**DOI:** 10.1155/2012/762804

**Published:** 2011-11-16

**Authors:** Yunyan Zhang

**Affiliations:** ^1^Department of Radiology, University of Calgary, Calgary, AB, Canada T2N 1N4; ^2^Department of Clinical Neurosciences, University of Calgary, Calgary, AB, Canada T2N 1N4; ^3^Hotchkiss Brain Institute, University of Calgary, Calgary, AB, Canada T2N 1N4

## Abstract

Multiple sclerosis (MS) is a complicated disease characterized by heterogeneous pathology that varies across individuals. Accurate identification and quantification of pathological changes may facilitate a better understanding of disease pathogenesis and progression and help identify novel therapies for MS patients. Texture analysis evaluates interpixel relationships that generate characteristic organizational patterns in an image, many of which are beyond the ability of visual perception. Given its promise detecting subtle structural alterations texture analysis may be an attractive means to evaluate disease activity and evolution. It may also become a new tool to assess therapeutic efficacy if technique issues are resolved and pathological correlates are further confirmed. This paper describes the concept, strategies, and considerations of MRI texture analysis; summarizes applications of texture analysis in MS as a measure of tissue integrity and its clinical relevance; then discusses potentially future directions of texture analysis in MS.

## 1. Introduction

Multiple Sclerosis (MS) is characterized by heterogeneous histopathology including inflammatory infiltrates, demyelination, remyelination, axonal damage, and gliosis [1]. Consequences of irreversible structural injury eventually lead to progressive physical disability and functional impairment [2]. T2 lesion number and volume are commonly used to evaluate disease activity and burden [3], which however are pathologically nonspecific and correlate only moderately with clinical outcomes. Accurate identification and quantification of pathological changes may facilitate a better understanding of disease pathogenesis and progression and help identify novel therapies for MS patients.

Structural abnormalities that appear regular may be extracted by visual inspection while complex patterns of pathology that are commonly encountered in medical images are difficult to interpret and require the employment of advanced analysis techniques [4]. As an emerging quantitative approach, texture analysis demonstrates promise to detect subtle structural alterations that are not perceivable on conventional magnetic resonance imaging (MRI). This paper describes the concept, strategies, and considerations of MRI texture analysis; summarizes the potential of texture analysis as a measure of tissue structural property and the clinical relevance; then discusses possible future directions of MRI texture analysis in MS.

## 2. The Concept

**Figure 1:**
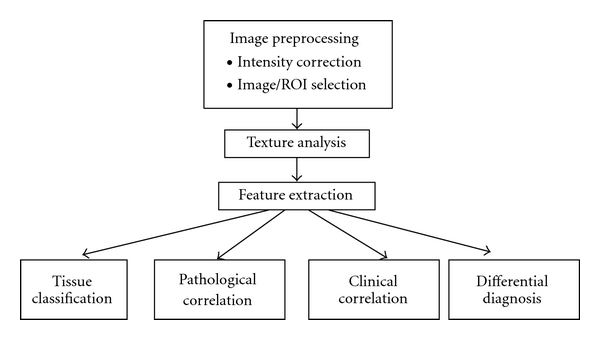
Overview of general texture analysis pipelines in the MRI of MS.

Texture analysis is an image postprocessing approach that extracts quantitative information from a digital image based on mathematical analysis: it can be applied to any image and is used in fields as diverse as medicine and geology [5–8] (Figure 1). A two-dimensional (2D) MR image is a digitized picture of elements (pixels), characterized by spatial location and gray-level intensities. MRI texture analysis evaluates the organizational pattern of image pixels that is unique to the underlying substrates in a tissue. Texture features are, in fact, mathematical parameters that describe the distribution of gray-level intensities to reflect the structural regularity of the imaged tissue [9]. Consequently the structural property of a tissue determines the nature of its texture, which is inevitably affected by histopathological development. Intuitively, texture can be described as smooth or rough, regular or irregular, fine or coarse.

## 3. Approaches

Numerous strategies have been proposed for the examination of image texture. Depending on the particular method used to assess interpixel relationships, a variety of dedicated texture features can be derived. Both statistical and spatial frequency-based approaches are used preferably in neurological imaging applications including MS, which are described below (Table 1). Note that the mathematical basis, calculation procedure, and feature extraction strategies of these texture analysis methods have been discussed comprehensively in previous publications and are not discussed in detail in this paper.

### 3.1. Statistical Approach

This approach attempts to characterize image texture using statistical parameters. First-order statistics (i.e., mean and variance of gray level, mean and variance of gradient) provides a global assessment of pixel distribution and is relatively intuitive and self-explaining. Second- or higher-order statistics characterizes local texture properties in an image which is based primarily on the cooccurrence matrix (GLCM) [7] and run-length matrix (RLM) [10].

**Figure 2:**
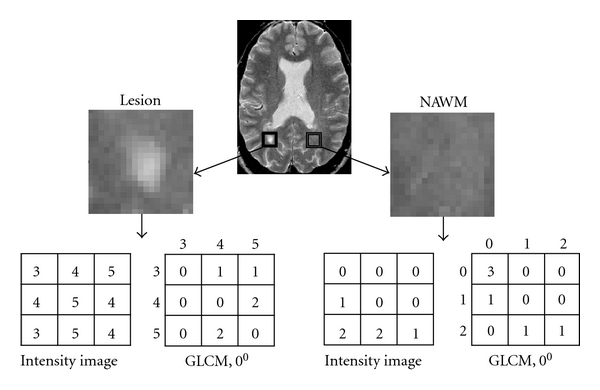
Schematic demonstration of texture analysis in the T2-weighted MR image from an MS patient (top) using a statistical method (gray-level cooccurrence matrix, GLCM). Areas of a focal lesion (box in the right) and the contralateral NAWM are delineated respectively, where texture analysis is conducted. Shown are different GLCMs computed at 0 degree based on the small sample subregions from the lesion (bottom right) and the NAWM of the patient.

The GLCM method has been investigated heavily since its first introduction by Haralick et al. in 1973 [7] and has demonstrated considerable promise in MRI texture analysis. In brief, the GLCM method computes the joint probability of two pixels which have particular (cooccurring) gray-level values, with a distance *d* (*d* = 1,…, *n* pixels in a dimension) apart along a given direction (0, 45, 90, and 135 degrees) (Figure 2). Fourteen parameters can be derived from each GLCM, which collectively reflect the homogeneity, local contrast, correlation, and complexity of an image.

The RLM method [9, 10] quantifies image texture in a similar manner as the GLCM approach. Basically, a gray level run dictates the number of times two or more pixels having the same value in a preset direction, and the RLM is the matrix of run-length frequency occurring in an image in each (generally 4) direction considered. Features derived from RLM represent fine (long runs) or coarse texture (short runs) of an image.

### 3.2. Spatial Frequency-Based Approach

Pixel patterns that create a unique texture in an image also generate a unique frequency distribution at the spatial scale of the pattern. Specifically, fast changing gray level values represent high frequency content. Conversely, low frequencies relate to slow changing or no change gray-level values [9]. Ideally, the frequency content held in an image can be calculated using the Fourier transform (FT). However, the FT cannot isolate frequency profiles specific to individual spatial locations [11, 12]. Moreover, the determination of slow or fast gray-value changes also depends on the scale that is utilized to examine the image. For instance, an image area would demonstrate little variations if analyzed by a large scale (or from a far distance) whereas an area would yield detailed information if identified by a small scale (or from a close distance).

**Figure 3:**
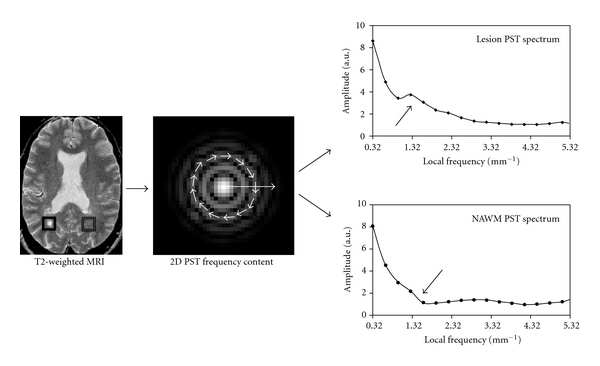
Texture analysis of the same image demonstrated in Figure 2 using a spectral method (polar Stockwell transform, PST). The PST spectra were first calculated as radius and orientation in polar coordinate (middle figure with arrows in circle); then a 1-dimentional spectrum (texture curve) was obtained by integrating frequencies along the radial direction for each frequency and pixel. Plots in the right side of the figure demonstrate the average texture curve of central 5 × 5 pixels in the lesion (right box) and the contralateral NAWM of the patient. Note that the amplitude of the low-frequency range appears greater (coarser) in the lesion than in the NAWM (arrows).

A few advanced transforms have been developed trying to characterize localized frequencies specific to each pixel location. One of the versatile approaches is the wavelet transform (WT) which characterizes multiscale frequency content (i.e., wavelet coefficients) at each spatial location of an image [13]. This flexibility allows the WT to decompose image texture scale specifically which is ideal for a tissue of heterogeneous pathology. Nonetheless the WT is computationally expensive and lack of intuitive [8]; it has yet no direct applications in MS. The Stockwell transform (ST) is a recent advancement in spatial frequency analysis [11, 14]. While analog to the WT for its multiresolution ability, the ST is a Fourier-based analysis that provides unique frequency spectra (Figure 3) at all pixel locations of an image. Moreover, rotationally invariant information can be obtained by eliminating the angular variance of image texture using the polar form of ST (PST) [15], which is a gifted advantage as medical images are prone to movement artifacts (Figure 3).

## 4. Considerations

While texture analysis is potentially a new tool to identify subtle structural changes in a tissue, there are concerns regarding its robustness to the variations in imaging protocol and MRI scanner. In theory, differences in acquisition parameters, imaging sequences, and the homogeneity of radio-frequency (RF) field cause alterations in pixel arrangements and therefore MRI texture. This potential limit is drawing much attention and has partially confounded the clinical use of texture analysis. On the other hand, few recent studies argued that MRI texture analysis is relatively tolerant to the imaging variables and support further investigation [16–18].

Mayerhoefer et al. [16] assessed the impact of variance in acquisition parameters (number of acquisitions, repetition time, echo time, sampling bandwidth, and spatial resolution) to texture indices and pattern discrimination. Texture features were increasingly sensitive to the variation of acquisition parameters in images with increasingly spatial resolution. However, the variation in imaging parameters had little impact on texture measurements if the image had sufficient spatial resolution. Meanwhile, GLCM-derived texture parameters outperformed the other statistical and WT ones. In another study of similar purpose, Harrison et al. [17] evaluated whether texture analysis was sensitive to image acquisition and processing protocols by assessing differences between three types of imaging sequences, two anatomical levels of interest, three sequential slices, and two methods of delineation of regions of interest (ROIs). A total of 280 statistical and WT-based parameters were extracted from lesions, WM regions further away from lesions (WM), WM regions adjacent to lesions (NAWM), cerebrospinal fluid (CSF), and basal ganglia of 38 patients with either MS or clinically isolated syndrome (CIS). The authors showed that MRI texture analysis provided an excellent distinction between tissues of interest (96–100% accuracy). There was no significant difference in the results of texture-based classification between imaging sequences, anatomical levels, or between temporal imaging slices within tissue. Moreover, Savio et al. [18] have studied the effect of slice thickness to texture analysis of MS lesions and the NAWM. The signal intensity of three 1-mm consecutive slices was averaged to simulate a 3-mm slice that is commonly utilized in clinical imaging. There were moderate differences in the distribution of texture values between 1-mm and 3-mm slices, which nonetheless did not compromise the classification results (lesion versus NAWM) even using different slice thickness between training and test datasets.

The robustness of texture analysis seems to be indicated by these investigations. However, the impact of imaging factors was assessed based on the accuracy of texture-associated classifiers. While it is a popular approach in computer-assisted diagnosis, the classification step may have complicated the evaluation process and added difficulty for interpretation. Direct comparison of texture characteristics may be helpful in the future to clarify this issue. Furthermore, it has not been fully investigated whether texture measures are portable between imaging centers; some researchers [19, 20] suggest that the use of test objects at different MRI scanners may be a practical means for feature normalization.

## 5. Applications

### 5.1. Characterization of Structure Properties

While pathologically different, the activity of many MS lesions cannot be distinguished on conventional MRI based on their appearances. Using a texture-based segmentation approach, Yu et al. [21] examined the feasibility of texture analysis to differentiate activity levels of 32 lesions from 8 relapsing-remitting (RR) MS. Forty-two features were extracted from first- and higher-order (GLCM and RLM) statistics from each lesion. By referencing the status of gadolinium (Gd)-enhancement, texture analysis based on T2-weighted MRI (T2W) allowed an accurate classification of both active (88%) and nonactive lesions (96%). As an exception to most texture analysis studies, features from RLM demonstrated better discrimination potential than that from GLCM. In addition, new evidence showed that a texture-based pattern recognition system was able to differentiate MS lesions from other neurological abnormalities on MRI [22].

Despite the dominance of multifocal pathology, diffuse abnormalities are observed in the normal appearing white matter (NAWM) and gray matter (NAGM) of MS patients [23]. Recently, J. Zhang et al. [24] assessed the texture property of MS lesions, NAWM, and normal WM from 16 patients and 16 controls and compared the segmentation ability of joint statistical and spectral features (>200) with that of GLCM features alone. The classification accuracy based on combined sets of texture parameters was superior (100%) compared to GLCM features only between MS lesions and NAWM or normal WM; however, the classification power was compromised (58.33%) in an attempt to differentiate NAWM from normal WM. The disadvantage of statistical texture analysis to detect NAWM abnormality was also implicated in a previous study [25], which compared the texture of frontal NAWM in 41 MS patients with the texture of normal WM at the same region of the brain in 10 controls. No texture difference was identified, which was presumably due to limited resolution of MR images.

Great potential of texture analysis has also been demonstrated using spectral features derived from the PST. In a longitudinal study, Y. Zhang et al. [26] analyzed the texture of new enhancing lesions in comparison with chronic lesions and the NAWM on T2W MRI from 10 RRMS patients. The texture in contrast-enhancing lesions was significantly coarser than that in chronic lesions or the NAWM on the same imaging slice; it improved gradually thereafter only in the acute lesions. The texture in chronic lesions remained relatively stable over time, as did the texture in the NAWM. This study also showed that the recovery of acute lesions seemed to be associated with the degree of coarse texture during enhancement. Using a similar method, Y. Zhang et al. [27] investigated whether texture was different between new acute T1 hypointense lesions (acute black holes, ABHs) that persist and those that resolve over time. The ABHs were classified as transient (tABHs), suggesting repair [28], or persistent (pABHs), reflecting destruction [29] based on their eventual T1 MRI appearance (isointense or hypointense) 5–8 months later. The tABHs demonstrated significantly finer texture than the pABHs when first seen on T1W MRI. These intriguing data suggest that inflammatory demyelination generates a heterogeneous signal manifesting as coarse texture, whereas organized tissue generates a homogeneous signal manifesting as fine texture (the texture of repairing tissue or the NAWM).

### 5.2. Clinical Relevance

The demonstration of clinical relevance is important for a potential imaging measure as part of the validation process. Mathias et al. [30] performed texture analysis on T2W MRI of the spinal cord from 40 MS patients (10 each of RRMS, primary progressive MS, secondary progressive MS, and benign MS) and 10 controls. Eight texture features (4 first-order and 4 GLCM statistics) were extracted from the entire cross-sectional area of the segmented spinal cord. Mean texture features were significantly different between all patients and normal controls and between normal controls and patient subgroups except for benign MS. In particular, while not all significant, MRI texture was generally coarser (i.e., increased entropy and decreased angular second moment) in MS patients than in controls, which was identified before detectable spinal cord atrophy. Moreover, two texture features (mean gradient and mean gray level) correlated significantly with disability as assessed by the Expanded Disability Status Scale (EDSS) in MS subjects. The authors suggested that texture analysis might be a potential tool to monitor changes associated with patient disability; however, the reproducibility and sensitivity of texture measurements must be improved. Similarly, by analyzing the GLCM texture of magnetization transfer ratio (MTR, a potential measure of tissue injury) maps of the brain from 23 controls, 38 patients with CIS and 35 patients with MS, Tozer et al. [31] found that the texture in MS differed from CIS and controls but did not differ between the later two groups. Furthermore, GM MTR texture correlated with neurological disability and WM MTR texture was associated with cognitive measures in MS. In the future, more studies of texture analysis for its capacity to characterize GM pathology would be desirable as conventional MRI is suboptimal to define GM plaques, which however demonstrated considerable pathological and clinical relevance [32, 33].

In a more recent study, Loizou et al. [34] assessed the texture of MS lesions and the NAWM from 38 patients with CIS and normal WM from 20 volunteers and examined the relationship between texture severity of MS lesions and disease progression over approximately 2 years. T2 MRI texture was analyzed using an amplitude modulation-frequency modulation (AM-FM) method, a technique with similarities to the multiscale spectral analysis. Consisting with other published reports, there was significant texture difference between lesions, NAWM, and normal WM. More interestingly, lesions of coarser texture at baseline associated with more severe disability (EDSS > 2) accumulated over 2 years while lesions of finer texture at baseline were found in patients who developed less disability (EDSS ≤ 2). The AM-FM classifier based on medium frequency instantaneous amplitude (relatively coarse texture) provided the best segmentation results to these lesions (area under the ROC curve = 0.76). The AM-FM features were proposed as potential measures of lesion load and disability progression in MS patients.

## 6. Future Directions

### 6.1. Pathological Correlates

It is hypothesized that pathological processes induce ultrastructural changes on the nanometer to micrometer scale, which manifest as pixel pattern changes on the millimeter scale of MR images. While it is not validated in human subjects, pathological correlates of texture analysis has been demonstrated in animal models of MS. Y. Zhang et al. [35] analyzed the texture of MS-like lesions on 7T T2W MRI in the spinal cord of mice with experimental allergic encephalomyelitis (EAE, a model of MS) using the PST. Increased PST texture was observed in EAE lesions compared with the control tissue, which corresponded with inflammation and demyelination. Moreover, texture analysis was evidenced to detect remyelination in a cuprizone mouse model of demyelination [36]. In that study, each mouse was fed either with cuprizone to induce demyelination or with normal diet as controls for 8 weeks. Texture analysis was performed before (day 0) and 13, 29, 32, 41, and 56 days on treatment. Yu et al. [36] showed that texture discrimination functions classified myelinated (day 0) and demyelinated (day 56) brain regions with near-perfect accuracy (95% and 98%, resp.). Furthermore, one of the texture parameters from the RLM, the horizontal gray-level nonuniformity (HGLNU), varied in concordance with changes in the myelination status of four brain regions: the HGLNU improved when reported remyelination occurred. While this study was limited by correlating indirectly with histological data inferred from literature, texture analysis appears to be a potential measure of myelin integrity. Nonetheless, further validation in MS subjects is necessary for a better understanding and interpretation of texture measurements in a clinical context. As a common validating system for MRI techniques, postmortem samples could be a great candidate; in that way, pathological specificity of texture analysis can be also investigated.

### 6.2. Evaluating Treatment Impact

Given its potential as a sensitive measure of tissue integrity and the clinical relevance, MRI texture analysis may be used to evaluate treatment impact in both overt MS lesions and in the disease prone NAWM. In this way, an integrated picture of therapeutic efficacy can be obtained beyond the detectability of lesion number and volume. In a feasibility study of 5 RRMS patients with active lesions, Y. Zhang et al. [37] investigated the texture of acute and chronic MS lesions and the NAWM before and after minocycline treatment using the GLCM method. Compared with inactive lesions and the NAWM, active lesions showed the greatest texture abnormality and exhibited the largest texture changes over 6 months, although statistical analysis was not significant. Recently, the promise of MRI texture analysis to identify treatment effect has been demonstrated in patients with non-Hodgkin lymphoma; [38] however, this is subject to further investigation in MS subjects.

## 7. Conclusions

MRI texture analysis demonstrates great potential in the study of MS. As an image processing strategy, texture analysis shows promise to extract clinically meaningful information from routine conventional MRI. Given its clinical relevance, texture analysis may be an attractive means to characterize disease activity and progression. However, the robustness of texture analysis to imaging protocols must be clarified before a significant clinical effect can be established. Furthermore, despite the promising outcomes in animal models, pathological correlates of texture analysis are subject to confirmation in MS subjects, whereby the role of texture analysis can be further tested as a potential tool of evaluating treatment benefits for MS patients.

## Figures and Tables

**Table 1:** Overview of common texture analysis approaches in MS.

	Assessment	Utility
Statistical approach		

First-order	Global assessment of pixel distribution	Self-explanatory yet lack of detail
Second-order		
Gray-level cooccurrence matrix (GLCM)	Joint probability of two pixels having cooccurring gray level values at a given distance and direction	Multiple properties of a texture (coarseness, correlation, contrast), less sensitive to larger scales
Run length matrix (RLM)	The number of times two or more pixels having the same value in a preset direction	Several properties of a texture (coarseness), less sensitive to larger scales

Spectral approach		
Fourier transform	Entire frequency profile, using sinusoid basis functions	Useful for signals without temporal changes
Wavelet transform	Scale-based frequency content, using a deformable localizing “mother” wavelet as basis function	Multiscale analysis; less intuitive and can be computation-expensive
Stockwell transform	Scale-based frequency content, using fast Fourier transform and a flexible Gaussian localizing window	Fourier-based multiscale frequency content; computation time varies by image size and algorithm
